# Mitochondrial A12308G alteration in tRNA^Leu(CUN)^ in colorectal cancer samples

**DOI:** 10.1186/s13000-015-0337-6

**Published:** 2015-07-19

**Authors:** Fawziah MA Mohammed, Ali Reza Rezaee khorasany, Elaheh Mosaieby, Massoud Houshmand

**Affiliations:** Medical Laboratory Sciences, Faculty of Allied Health Sciences, Kuwait University, Kuwait; Department of Pharmacodynamy and Toxicology, School of Pharmacy, Mashhad University of Medical Sciences, Mashhad, Iran; Department of cellular and molecular biology, Mazandaran university, Babolsar, Iran; Department of Medical Genetics, National Institute of Genetic Engineering and Biotechnology (NIGEB), Tehran, Iran

**Keywords:** Colorectal cancer, Mutation, Mitochondrial tRNA^Leu(CUN)^, A12308G mutation

## Abstract

**Background:**

Colorectal cancer is the third most common type of cancer in men and women and the second leading cause of cancer-related deaths in the United States and UK. Colorectal cancer is strongly related to age, with almost three-quarters of cases occurring in people aged 65 or over. Pre-symptomatic screening is one of the most powerful tools for preventing colorectal cancer. Recently, the use of mitochondrial tRNA genes mutation or polymorphism patterns as a biomarker is rapidly expanding in different cancers because tRNA genes perform several functions including processing and translation which are essential components of mitochondrial protein synthesis. The aim of the present study was to find out the association of mitochondrial A12308G alteration in tRNA^Leu(CUN)^ in colorectal cancer and its usage as a new biomarker screening test.

**Methods:**

A tumor tissues from 30 patients who had colorectal cancer were selected randomly. The A12308G alteration in tRNALeu (CUN) was screened in the 30 colorectal tumor tissues. For comparison, 100 blood samples of healthy controls using PCR-sequencing methods were selected and the following results were found.

**Result:**

The A12308G, a polymorphic mutation in V-loop tRNA^Leu(CUN)^, was found in 6 Colorectal tumor tissues and 3 healthy controls. A statistical significant difference was found between cases and control regarding the association of the A12308G mutation with the colorectal tumor (P < 0.05).

**Conclusions:**

The A12308G, a polymorphic mutation in V-loop tRNA^Leu(CUN)^, could be considered as pathogenic mutation in combination with mitochondrial external conditions and other mitochondrial genes in developing different diseases especially cancers and could be used as one of the diagnostic tool. Also it seems that maybe there is relevance between A12308G mutation and other mutations that it can cause various phenotypes.

**Electronic supplementary material:**

The online version of this article (doi:10.1186/s13000-015-0337-6) contains supplementary material, which is available to authorized users.

## Background

Worldwide, colorectal cancer (CRC) is the fourth most common cancer and affects both men and women equally and the American Cancer Society estimated that ~56,730 would die from this disease. The substantial mortality associated with this cancer makes it the leading cause of gastrointestinal cancer deaths [1]. Colorectal cancer is an uncontrolled cell division of the colon or rectal cells starting in the inner most layer and can grow through some or all of the other layers. These cells may also invade and destroy the tissue around them and spread to form new tumors in other parts of the body. Unfortunately, some colorectal cancers might be present without any signs or symptoms and often diagnosed late when the disease becomes more advanced. For this reason, it is very important to have regular colorectal screening tests for early detection when the disease is easier to cure/control. Screening has been found to be effective in reducing the incidence and mortality of colorectal cancer through the detection and removal of pre-cancerous lesions and through the detection of CRC in its early stages. Colonoscopy, sigmoidoscopy, and fecal occult blood tests are all recommended screening tests that have widespread availability [2]. Recently, Genetic testing is developed that offer more reliable options for colorectal cancer screening. Mitochondria play a central role in the regulation of cellular function, metabolism, free radical generation, and cell death. Defects in mitochondrial function have been speculated to have an impact on the development and progression of cancer [3].

Cancer development involves the accumulation of genetic changes that will happen in both nuclear and mitochondrial genes. In cancer cells, mutations in mtDNA were more readily detectable and 10 times abundant than nuclear DNA (nDNA), possibly due to the lack of introns, lack of histone protection, low efficiency of mtDNA repair systems and close proximity to damaging reactive oxygen species (ROS) [4–7].

Alterations in mitochondrial DNA (mtDNA) in the D-loop region as well as in other parts of the mitochondrial genome, including point mutations, deletions, insertions and genome copy number changes, are believed to be responsible for carcinogenesis in a variety of human cancers such as ovarian, colon, thyroid and endometrial cancer, salivary glands, liver, lung, gastric, brain, bladder, kidney, prostate, head and neck, breast cancer and leukemia [8–19].

Mutations in the Mt-tRNA genes have impact on the secondary and tertiary tRNA structure, and may cause transcriptional and translational defects and mitochondrial respiratory chain dysfunction consequently. More than half of mitochondrial mutations have been located in mt-tRNA genes which are hot spots for mitochondrial pathogenesis [20].

Therefore, mtDNA mutation pattern is a great molecular cancer biomarkers and it could increase the specificity of cancer detection and prediction. Here, we are studying about the human mitochondrial A12308G alteration in tRNA^Leu(CUN)^ in tumoral tissues from colorectal cancer patients.

## Methods

Tumor tissues samples, from thirty Iranian colorectal cancer patients were collected at the cancer institute of Imam Khomeini Tehran hospital. For comparative purposes, blood samples from100 healthy controls of matched age and sex were collected too. The DNA from tumoral tissues was extracted using QIAamp DNA FFPE kit (QIAGENE) while, DNA from blood samples obtained from healthy control was extracted using DNA fast kit (Genefanavaran, Tehran, Iran). The A12308G alteration in tRNALeu(CUN) was screened by sequencing the PCR products from both patients and control samples. Primer sequences are as described in Table 1 or Additional file 1. PCR was carried out in a total volume of 25 μl, containing 2.5 mM Mgcl2, 200 μM of each dNTP, 10 Pm of each primer, 100 ng total DNA and 1U taq DNA polymerase in thermal cyclers (Eppendrof, Master cyclers, 5330). Thermocycling conditions were 94 °C for 5 min, followed by 32 cycles of 95 °C for 1 min, annealing for1 min at 50 °C and extension at 72 °C for 45 s, and finally 72 °C for 10 min for 32 cycles. The PCR products were examined for specificity using 1.5 % agarose gel electrophoresis. Double-stranded automated sequencing was performed using an ABI 3100 sequencing machine (Applied Biosystems, Kavosh Fanavaran Kawsar Company, Iran). All fragments were sequenced in both forward and reverse directions. Sequence of tumoral tissues were analyzed using a Finch TV program (chromatogram viewer which displays DNA sequence traces) and compared to the Human Mitochondrial Reference Sequence NC_012920 provided by the National Center for Biotechnology Information (NCBI). The Chi-square test was used with SPSS (Statistical Package for the Social Sciences, version: 13) to examine the association between the presence of mutation/polymorphisms in colorectal tumoral tissues and the blood of healthy controls. P values < 0.05 were regarded as statistically significant.

**Table 1:** Mitochondrial Primers for PCR-Sequencingof tRNA^Leu(CUN)^

Name	Primer	Sequence
ONP71	L.F11901-11920	5’-TGCTAGTAACCACGTTCTCC-3’
ONP46	H.R12420-12401	5’-TTTGTTAGGGTTAACGAGGG-3’

## Resutls

Homoplasmic A12308G, a polymorphic mutation in V-loop (tRNA^Leu(CUN)^), was found in 6 colorectal tumor (20 %) and 3 healthy controls (3 %). This difference is statistically significant (P = 0.05).

## Discussion

Various human diseases have been associated with mtDNA mutations, indicating that dysfunction of the components of oxidative phosphorylation encoded by the mitochondrial genome can be deleterious [21]. Abnormalities in mtDNA have proven to be associated with leber’s hereditary optic neuropathy (LHON) [22], Primary open-angle glaucoma (POAG) [23, 24], pseudoexfoliation glaucoma (PEG), primary angle closure glaucoma (PACG), other spontaneous optic neuropathies [25–27] and male infertility [28]. Moreover, 25-80 % of somatic mutations in mitochondrial DNA are found in various neoplasms [29]. Also, in 2012 the role of the mitochondrial tRNA genes was analyzed in patients with asthma compared with a set of healthy controls. They suggested that the mitochondrial tRNA genes play a key role in asthma development [30]. The use of mtDNA mutation patterns as a biomarker is rapidly expanding in rare metabolic diseases, aging, cancer, tracing of human migration patterns, population characterization and human identification in forensic science [31]. It seems that the mitochondrial genome is more useful in detecting tumor cells in body fluids and cytological specimens than mutations in nuclear DNA had been confirmed.

In the present study, to the best of our knowledge, this is the first reported association between colorectal cancer and mtDNA A12308G alteration in tRNALeu(CUN). The A12308G change was introduced as a common polymorphism by Houshmand at the first time [14]. Several studies described the association of mt-tRNA mutations with human cancers. This mutation came to the attention of the breast cancer research communities as a plausible candidate marker for increased breast cancer susceptibility [29, 32]. In USA, the A12308G polymorphism was introduced as an important factor in kidney and prostate cancer risk [16]. In India, the A12308G mutation was seen as a significant change in the risk of oral cancer [33]. This alteration was, also, reported as a multiplier risk factor in advanced breast cancer tumors in European – American patients [34]. Increased prevalence of the A12308G mutation in mitochondrial tRNA^Leu(CUN)^gene associated with Friedreich's ataxia in Iran, was reported [35]. In previous studies, A12308G alteration has occurred in association with another disease causing alteration in MELAS, myopathy and primary congenital glaucoma (PCG) where three such changes (G10398A, A12308G and G13708A) were present in the later [36, 37]. Moreover, the A12308G polymorphism in tRNA^Leu(CUN)^ increases the risk of developing stroke in patients with the A3243G mutation [38]. So, this polymorphism may act as a secondary mutation in this disease pathogenicity. The A12308G variation is also associated with increased ROS production [39]. Nine main European haplotypes (H, I, J, K, T, U, V, W and X) were analyzed in a series of patients with prostate and renal cancers studied by Booker et al. Using the A12308G substitution in tRNA^Leu^ as a marker of the mtDNA haplogroup U, it was found that patients carrying this haplogroup had an increased risk of renal and prostate cancer [16]. Some studies showed an increased frequency of the A12308G substitution in mitochondrial patients carrying mtDNA single macrodeletion. In this group of patients, A12308G substitution is associated with a higher relative risk of developing pigmentary retinal degeneration, short stature, dysphasia–dysarthria and cardiac conduction defects [40]. Moreover, the A12308G was found in 8 Alzheimer’s disease patients [41]. In the case of endometrial adenocarcinoma the presence of mitochondrial A12308G alteration in tRNA^Leu(CUN)^ was reported [42, 43]. Study in Italy stated that Mitochondrial DNA mutations have been causally linked with cardiomyopathies, both dilated (DCM) and hypertrophic. They identified the T12297C mutation in the mtDNA-tRNA^Leu(CUN)^ of a patient diagnosed with DCM. In the variable loop of the same tRNA, their patient also carried the A12308G transition [44].

## Conclusions

In conclusion, the present study revealed that mitochondrial research will enable to establish biomarkers helping to identify individuals at high risk for developing specific cancer types and to develop screening approaches for early diagnosis of cancer. In addition, it seems that more research is essentially needed to understand the effect and role of the A12308G mutation as a common polymorphism or an inherited predisposition factor in the carcinogenesis. We believe that this mutation associated with other mutations and/or factors would lead to diverse phenotypes.

### Consent

Written informed consent was obtained from the patients for the publication of this report and any accompanying images.

## Additional file

Additional file 1: Table 1. Mitochondrial Primers for PCR-Sequencing of tRNA^Leu(CUN)^.
